# Translating and Adapting the DISCERN Instrument Into a Simplified Chinese Version and Validating Its Reliability: Development and Usability Study

**DOI:** 10.2196/40733

**Published:** 2023-02-02

**Authors:** Yi Shan, Zhaoquan Xing, Zhaogang Dong, Meng Ji, Ding Wang, Xiangting Cao

**Affiliations:** 1 School of Foreign Studies Nantong University Nantong China; 2 Department of Urology Qilu Hospital of Shandong University Jinan China; 3 Department of Clinical Laboratory Qilu Hospital of Shandong University Jinan China; 4 School of Languages and Cultures The University of Sydney Sydney Australia

**Keywords:** DISCERN, translation, adaptation, validation, quality, patient-targeted health information, treatment choice

## Abstract

**Background:**

There is a wide variation in the quality of information available to patients on the treatment of the diseases afflicting them. To help patients find clear and accessible information, many scales have been designed to evaluate the quality of health information, including the Patient Education Materials Assessment Tool; the Suitability Assessment of Materials for evaluation of health-related information for adults; and DISCERN, an instrument for judging the quality of written consumer health information on treatment choices. These instruments are primarily in English. Few of them have been translated and adapted into simplified Chinese tools for health information assessment in China.

**Objective:**

This study aimed to translate and adapt DISCERN into the first simplified Chinese version and validate the psychometric properties of this newly developed scale for judging the quality of patient-oriented health information on treatment choices.

**Methods:**

First, we translated DISCERN into simplified Chinese using rigorous guidelines for translation and validation studies. We tested the translation equivalence and measured the content validity index. We then presented the simplified Chinese instrument to 3 health educators and asked them to use it to assess the quality of 15 lung cancer–related materials. We calculated the Cohen κ coefficient and Cronbach α for all items and for the entire scale to determine the reliability of the new tool.

**Results:**

We decided on the simplified Chinese version of the DISCERN instrument (C-DISCERN) after resolving all problems in translation, adaptation, and content validation. The C-DISCERN was valid and reliable: the content validity index was 0.98 (47/48, 98% of the items) for clarity and 0.94 (45/48, 94% of the items) for relevance, the Cronbach α for internal consistency was .93 (95% CI 0.699-1.428) for the whole translated scale, and the Cohen κ coefficient for internal consistency was 0.53 (95% CI 0.417-0.698).

**Conclusions:**

C-DISCERN is the first simplified Chinese version of the DISCERN instrument. Its validity and reliability have been attested to assess the quality of patient-targeted information for treatment choices.

## Introduction

### Background

A total of 80% of patients actively seek information on dealing with their health issues [1]. As reported in the literature, 25% of patients turn to books and leaflets for health information, and 33% seek information on the internet, although most of them regard their physicians as the most essential health information source [1]. Easy access to a variety of information enables patients to make medical decisions actively [2]. Only clearly stated and easily accessible information about prevention and treatment choices can facilitate patient adherence to professional health care providers’ recommendations and interventions. However, there is a wide variation in the quality of information available to patients on the treatment of the health problems afflicting them [3].

The quality of health and medical information is crucial in the context of patients gaining increased access to such information [2]. The quality of information does not merely depend on whether it is evidence-based (reliability), clear and accessible to patients (clarity and accessibility), and the latest or most recent information (timeliness) [2,4]. The quality of information also relates to potential conflicts of interest, the qualifications of information providers, and clear mention of other patient information sources [3]. Given that these factors considerably affect the quality of information, it is of paramount importance to provide patients with easily accessible, accurate health information to promote balanced, well-informed decisions and improve their health outcomes. Good-quality written consumer health information on treatment choices is accurate and based on the best and most up-to-date scientific evidence [5]. Currently, there is a lot of written consumer health information about treatment choices available from various sources. Not all such information is of good quality, and merely a small percentage is based on solid evidence [5]. Many publications available provide inaccurate or confusing advice [5]. This context may warrant the development of tools for assessing the quality of consumer health information. Such tools can safeguard patients from inaccurate or confusing information, making it relatively easy for them to decide which information to use and which to discard [5].

It has been estimated that >500 assessment tools have been developed for evaluating the quality of health information [4], including the Patient Education Materials Assessment Tool (PEMAT) [6]; the Suitability Assessment of Materials (SAM) for evaluation of health-related information for adults [7,8]; and DISCERN, an instrument for judging the quality of written consumer health information on treatment choices [9], among many others. These instruments have different focuses. The SAM assesses the suitability of health-related materials for adults, for example, the suitability of web-based sources of information on male infertility [10] and the suitability of articles published by health-related WeChat public accounts [11]. The PEMAT evaluates the comprehensibility and actionability of health education materials [2,9] to identify problems with such materials. For example, Yiu et al [12] assessed web-based educational materials for patients who took non–vitamin K oral anticoagulants. Drawing on the PEMAT, Jamil et al [13] developed and improved “an integrated diabetes-periodontitis nutrition and health education module.” Both the SAM and the PEMAT can be used to appraise written, video, and audiovisual materials. Unlike the SAM and the PEMAT, DISCERN was designed to evaluate written consumer health information. It is oriented toward judging the quality of written consumer health information on disease treatment options rather than on general health education. We have developed the Chinese versions of the PEMAT and SAM in the other 2 studies contributed to *JMIR*. In recognition of the need for a general set of quality criteria for written consumer health information on treatment choices [5] in China, this study aimed to develop the simplified Chinese version of DISCERN to facilitate “shared decision-making and evidence-based consumer choice” [5] with respect to treatment options.

DISCERN is a tool designed to aid users of consumer health information in judging the quality of written information regarding treatment choices [5]. Good-quality written information will help patients understand their treatment, know what to expect from the treatment, and choose the option that is best for them [5]. DISCERN is suitable for anyone who uses or produces information on treatment choices [5]. It can be used diversely as (1) an aid for individuals who are making decisions on treatment, (2) a screening tool for health information providers, (3) a checklist for authors and producers of written consumer health information, and (4) a training tool for health professionals to improve communication and shared decision-making skills [5]. A newly developed Chinese version of DISCERN is likely to be used in these 4 scenarios.

The original English version of DISCERN has been applied in many studies. McCool et al [3] applied DISCERN to evaluate the quality of patient information on eczema. Mueller et al [14] examined the content-related quality of atopic dermatitis videos and their perception among YouTube users. Sun et al [15] assessed the quality of information on liver diseases provided in Wikipedia and Baidu Encyclopedia. Yacob et al [16] investigated the quality of Wikipedia articles and their corresponding chapters in a standard undergraduate medical textbook on surgery. Carlsson and Axelsson [17] studied the quality of web-based patient information on medically induced second-trimester abortions. Cuan-Baltazar et al [18] rated the quality of web-based information on COVID-19. Kwakernaak et al [19] determined the quality of the websites that patients visited to find the right diagnoses. Memon et al [20] assessed the quality of web-based health information on the 10 most common fractures. Mueller et al [21] evaluated the quality of psoriasis-related videos. Although well-developed assessment instruments have been used for evaluating the suitability of health education materials in English-speaking societies, such scales are rarely applied in Chinese-speaking communities [22].

Although DISCERN was used in German-, French-, Dutch-, Chinese-, and Iranian-speaking communities [23-27], it has mostly been adopted in English-speaking countries [3]. It has been translated and adapted into Brazilian Portuguese [28], Spanish [29], and German [30] and validated in these languages, but no Chinese version of DISCERN is currently available in Chinese-speaking societies. We need to develop a simplified Chinese version of DISCERN for the following reasons: (1) very few English-language health information assessment scales have been translated and adapted into simplified Chinese tools; (2) patients seeking information on the treatment of health problems are confronted with a wide variation in the quality of the information available to them [3]; and (3) worse still, merely a small proportion of abundant consumer health information on treatment choices is based on solid evidence [5], and many publications available provide inaccurate or confusing advice [5]. Translating existing instruments for use in different language studies is a rapid and practical approach to assessment in the absence of a tool [22]. As painstaking efforts and considerable time and cost investments are involved in developing new tools [31], Mohamad Marzuki et al [32] strongly recommended that established, accessible, and reliable tools should be adapted, validated, and recorded cross-linguistically. Therefore, it is imperative to translate quantitative tools into the language of the culture that is investigated for studies in which these instruments are used [33].

However, cross-cultural research studies frequently involve methodological problems primarily concerning the translation quality and the comparability of research results in different cultures and ethnic groups [34]. Literal translation cannot guarantee a cross-culturally valid scale. The values that a tool reflect and the meanings of its comprising constructs can vary from culture to culture [25]. It is even more challenging to adapt an instrument in “a culturally relevant and comprehensible form” while keeping its original meaning and intent unchanged [34]. Research instruments need to be validated and proven reliable in each culture that is studied to investigate the health care needs of people from different cultural backgrounds [35]. A valid translated version of an assessment tool must undergo a rigorous translation process to achieve semantic and content equivalence in cross-cultural research [22]. It is also essential to adapt tools cross-culturally, but evidence of the best approaches to cross-cultural adaptation is still lacking [36]. To make the original tool and the translated version equivalent, proper translation and appropriate adaptation are essential [26,36,37].

Lung cancer is the most fatal cancer worldwide, and it is the leading cause of cancer-related deaths, representing nearly 20% of all cancer-related deaths [38]. An estimated 2.09 million new lung cancer cases occurred worldwide in 2018, ranking first among all types of cancer [38]. Lung cancer incidence among men and women has rapidly increased in China in recent years, posing a great threat to people’s health [38]. The mortality rate of lung cancer in China is relatively high compared with that in most countries [39]. Lung cancer mortality in China is projected to increase by approximately 40% between 2015 and 2030 [40]. In addition to smoking cessation, reducing air pollution, and early detection and standard treatment, increasing public awareness is an important intervention for the prevention and treatment of lung cancer [41]. Although all patients are willing to read information on lung cancer [41], Chinese people lack general knowledge of lung cancer [41]. Over 80% of patients with lung cancer in China miss the optimal time for treatment, ending up generally presenting with terminal disease [42]. Given the critical issue of delayed diagnosis, it is imperative to raise public awareness of lung cancer to ensure effective prevention and early detection [41] and inform shared decision-making on treatment choices. A crucial part of increased consumer involvement in decision-making on treatment is access to good-quality information [5]. To this end, materials providing education on lung cancer are likely to play a crucial role. Such materials need to be assessed for quality before being delivered to the public for educational purposes considering the wide variation in the quality of the information currently available to patients on the treatment of health problems [3].

### Objectives

This study aimed first to translate DISCERN into a simplified Chinese version, then to adapt it to the Chinese language and culture, and finally to verify its validity and reliability for evaluating the quality of lung cancer–related health information on treatment choices written in simplified Chinese.

## Methods

### Overview

First, DISCERN was translated into a simplified Chinese version using rigorous guidelines for translation and validation studies. We then tested its translation equivalence and measured its content validity index. Subsequently, we presented the simplified Chinese instrument to 3 health educators from Qilu Hospital affiliated to Shandong University, China, and asked them to use it to assess the quality of 15 lung cancer–related materials. We calculated the Cohen κ coefficient and Cronbach α for all items and for the entire scale to determine the reliability of the new tool.

### The DISCERN Instrument

DISCERN is a validated scale to assess patient-targeted health information in print and digital texts. It comprises 16 questions (items) that mainly evaluate the aim of a publication; the relevance, accuracy, and timeliness of the information in the publication; treatment options and their effect on quality of life; and the merits, demerits, and side effects of different treatments [3,9]. These 16 items are grouped into 3 sections. Section 1 (items 1 to 8) concerns the reliability of the information in a publication, section 2 (items 9 to 15) addresses the quality of information on treatment choices, and section 3 (item 16) focuses on the overall rating of a publication as a source of information [9]. Items 1 to 15 are rated on a 5-point Likert scale: 1=No (not meeting the criterion), 3=Partially (partly meeting the criterion), and 5=Yes (meeting the criterion) [9]. Item 16 is rated on a 5-point Likert scale: 1=Low, serious, or extensive shortcomings; 3=Moderate or potentially important but not serious shortcomings; and 5=High or minimal shortcomings [9]. The total score that a publication can obtain ranges from 16 to 80. On the basis of the overall scores obtained, the quality of a publication is rated as very poor (16-26), poor (27-38), fair (39-50), good (51-62), and excellent (63-80) [16,43].

### Translation and Adaptation of DISCERN

#### Overview

**Figure 1:**
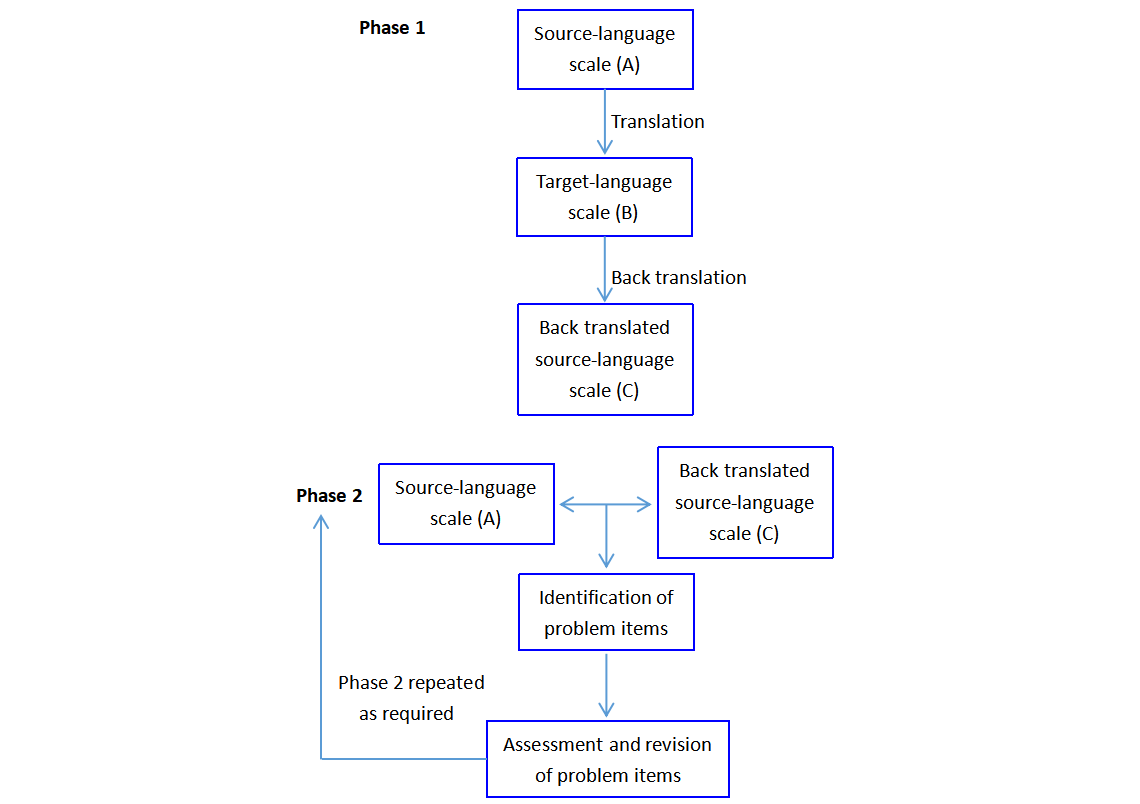
The entire process of developing the simplified Chinese version of DISCERN.

Considering these challenges, we translated and cross-culturally adapted the original DISCERN following established translation and adaptation guidelines in the literature, including forward translation, back translation, cultural adaptation, and translation equivalence testing [33,43-45]. It is essential to use multiple techniques in all cross-cultural studies [46]. The entire process of translation, translation equivalence testing, and revision of problem items is shown in Figure 1, which was taken from Sperber [34].

#### Forward Translation

The original DISCERN was forward translated into Chinese by a native Chinese speaker who was highly proficient in English. Specifically, what was translated included the section names, the 16 question items and their corresponding hints, and the responses to these 16 items.

#### Adaptation: Semantics Evaluation and Consolidation of the Translated Version

In this procedure, we intended to adapt the translated Chinese version linguistically and culturally and, thus, achieve idiomatic equivalence by checking semantics, pragmatics, wording, and grammar. In total, 2 qualified bilingual translators were requested to review the translated Chinese version independently to identify problematic items in linguistic and cultural appropriateness. Afterward, we held a panel meeting to discuss the results of the bilingual translators’ reviews with the native Chinese translator and modify the Chinese version. The discussion and revision focused on the linguistic and cultural appropriateness of the Chinese version, ensuring that the key concepts in the target version corresponded with the logic, language, and experiences of the target language and culture and that positive cultural images and examples were used in cross-cultural translation [7]. This is especially true when one version has ideas and words that appear socially insensitive or difficult to express in the other version [45] and when disparities exist between the cultural realities of the target and source languages. In summary, we meant to make the adapted Chinese version fulfill the following criteria: (1) faithfulness to the original version and linguistic expressiveness; (2) better adaptation of words, phrases, or sentences to simplified Chinese when the literal translation did not make much sense or no literal translation could be used; and (3) great importance to terms and expressions easily accessible to lay people with low health literacy, as targeted specifically by DISCERN [9].

#### Back Translation

In back translation, the revised Chinese version was translated back into the source language (ie, the original English) to verify the translation of the DISCERN instrument. Informed by Sperber [34], we carefully chose another qualified bilingual translator to perform back translation, and this translator had no previous knowledge of the original DISCERN to avoid recall bias, warranting the quality of the back translation [47,48]. The purpose of back translation was to find discrepancies between the original (source) version and the translated (target) version [34]. We were allowed to modify words or concepts without clear equivalent expressions in the target language [33]. We repeated back translation until we felt satisfied with the equivalence between the source-language version and the target-language version [49]. Without back translation, we cannot validate the adequacy of instrument translation [33].

#### Translation Equivalence Testing and Further Adaptation

Translation equivalence testing [50] was used to test the translation equivalence of the Chinese version. It was designed to modify the translated version by finding discrepancies between the original and translated versions. This step was achieved by comparing the back translated English version with the original English version in terms of the similarity of interpretability (SI) and comparability of language (CL). “Comparability of language refers to the formal similarity of words, phrases, and sentences. The similarity of interpretability refers to the degree to which the two versions would engender the same response even if the words are not the same” [50]. We invited a native English speaker to compare the 2 English versions (original and back translated) in terms of SI and CL. Informed by Chang et al [22], we used a 4-point Likert scale to rate these 2 English versions as *extremely similar*, *similar*, *not similar*, and *not at all similar* for SI and another 4-point Likert scale to rate these 2 English versions as *extremely comparable*, *comparable*, *not comparable*, and *not at all comparable* for CL.

A panel discussion was held to correct items rated with 3 or 4 by the native English speaker. The revised items were then retranslated and compared with the corresponding items in the original English version. We repeated such revision, retranslation, and comparison until we found the 2 English versions comparable and interpretable in nearly the same way. To achieve linguistic and cultural appropriateness, we asked another qualified bilingual translator to further improve the Chinese version by revising problematic items, if there were still any.

### Psychometric Testing: Verifying the Validity and Reliability of the Chinese DISCERN

Many instruments have been developed to assess health education texts [51-53], but few publications have described the psychometric properties of these tools [51]. In this study, we tested the psychometric properties of the newly developed simplified Chinese DISCERN, including its content validity and reliability.

#### Content Validation and Final Revision

Rubio et al [54] proposed asking a group of experts to provide constructive feedback on the quality of a newly developed tool and on the objective criteria for evaluating each of its items for content validation. In this study, we requested 3 Chinese health educators (ZD, DW, and CX) to assess the clarity and relevance of the 16 items of the further adapted Chinese version of DISCERN to validate its content. A 4-point Likert scale was once again adopted: *1=not clear*, *2=major revisions needed to make it clear*, *3=minor revisions needed to make it clear*, and *4=clear* for clarity and *1=not relevant*, *2=major revisions needed to make it relevant*, *3=minor revisions needed to make it relevant*, and *4=relevant* for relevance [22]. The content validity index was calculated by dividing the number of items that were rated with 3 or 4 by the total number of rated items [54].

When negatively rated, problematic items, if any, were further revised and improved in clarity and relevance by a highly qualified health educator (ZX) to increase the content validity index. The final simplified Chinese version of the DISCERN instrument (C-DISCERN) was developed in this step.

The 4 health educators all had a public health education background. Of them, 2 were highly qualified health educators (ZD and ZX) who are working as professors and physicians at Qilu Hospital affiliated to Shandong University, China, since they received their doctorate at Shandong University. The other 2 (DW and CX) are studying for their master’s degree in public health education at Shandong University, China. Their professional educational background and experience in engaging with patients at Qilu Hospital affiliated to Shandong University could qualify them for the content validation of the newly developed tool.

#### Reliability Testing

We verified the reliability of C-DISCERN by testing its interrater reliability and internal consistency.

##### Interrater Reliability

To validate the interrater reliability of C-DISCERN, we determined the scoring agreement between 2 health educators who were requested to use C-DISCERN to assess 15 print lung cancer–related health materials. We calculated the Cohen κ coefficient to ascertain the scoring agreement between the 2 raters by applying the measurement of Cohen κ coefficients proposed by Fleiss [55]: *<0.20=poor agreement*, *0.21 to 0.40=fair agreement*, *0.41 to 0.60=moderate agreement*, *0.61 to 0.80=strong agreement*, and *>0.80=nearly complete agreement* [55].

##### Internal Consistency

We asked 3 health educators to rate a lung cancer–related health brochure to validate the internal consistency of C-DISCERN by calculating the Cronbach α. To this end, we measured the Cronbach α for the entire instrument with a 95% CI. A Cronbach α of ≥.70 indicated the internal consistency of an instrument [56,57], and a value of ≤.20 implied the removal of an item or domain [58].

### Data Collection and Analysis

Digital scoring and rating sheets were used to document the collected data manually. After that, we used SPSS (version 22.0; IBM Corp) to process the data to measure the content validity index, Cohen κ coefficient for interrater agreement, and Cronbach α for internal consistency of C-DISCERN.

### Ethics Approval

This study was approved by the ethics review board of Qilu Hospital affiliated to Shandong University, China. The review number is KYLL-202208-026.

## Results

### Translation of DISCERN

#### Forward Translation and Corresponding Adaptation

After being adapted, the previously problematic items in C-DISCERN were far more appropriate both linguistically and culturally, although techniques such as addition, omission, substitution, and replacement were adopted to achieve loyalty to the original text, expressiveness of the target text, idiomatic expression of some target words or phrases, and readability and understandability of technical terms, as shown in Table 1.

**Table 1:** Adaptation of problematic items in the simplified Chinese version of the DISCERN instrument (C-DISCERN).

Category of adaptation	Problematic items in C-DISCERN marked through corresponding items in DISCERN	Before adaptation	After adaptation
Loyalty	4. Is it clear what sources of information were used to compile the publication (*other than the author or producer^a^*)? 9. Does it describe how each treatment works? HINT: ...^b^	“other than the author or producer” was omitted or neglected. “each” was omitted or neglected.	“other than the author or producer” was retained. “each” was retained.
Expressiveness	1. Are the aims clear? HINT: Look for *a clear indication* at the beginning of the publication of: ... 7. Does it provide details of additional sources of support and information? HINT: Look for suggestions for further reading or *for details* of other organizations providing advice and information about the condition and treatment choices. 12. Does it describe *what would happen* if no treatment is used? 14. Is it clear that there may be more than one possible treatment choice? HINT: Suggestions of *alternatives to consider or investigate further* (including choices not fully described in the publication) before deciding whether to select or reject a particular treatment choice. ...	“a clear indication” was translated into “indicate.” “for details” was translated literally. “what would happen” and “used” were translated literally. “alternatives to consider or investigate further” was translated literally.	“a clear indication” was translated into “the content explicating the following questions.” “for details” was omitted. “what would happen” and “used” were translated into “consequence” and “adopt,” respectively. “alternatives to consider or investigate further” was translated into “other alternatives of treatment choices.”
Idiomatic expression	3. *Is it relevant?* HINT: Consider whether: the publication addresses the questions that readers might ask. *recommendations* and suggestions concerning treatment choices are realistic or *appropriate*. 4. Is it clear what sources of information were used to compile the publication (other than the author or producer)? HINT: *Check* whether *the main claims or statements made about treatment* choices are *accompanied by* a reference to the sources used *as evidence,* eg, a research study or expert opinion. ... 8. Does it refer to *areas* of uncertainty?	“Is it relevant?” was translated literally. “check,” “the main claims or statements made about,” and “as evidence” were translated literally. “areas” was translated into “places.”	“Is it relevant?” was replaced by “Does the publication address the questions that readers might ask?” “check” was omitted, “the main claims or statements made about” was translated into “in the text/publication,” and “as evidence” was translated into “evidently.” “areas” was translated into “factors.”
Readability and understandability	8. Does it refer to areas of uncertainty? HINT: *Look for discussion of the gaps* in knowledge or differences in expert opinion concerning treatment choices. Be wary if the publication implies that a treatment choice *affects everyone in the same way,* eg, 100% *success rate* with a particular treatment. 12. Does it describe what would happen if no treatment is used? HINT: Look for a description of the risks and benefits of postponing treatment, of *watchful waiting* (ie, monitoring how the condition progresses without treatment), or of *permanently forgoing treatment.*	“look for discussion of the gaps,” “affects everyone in the same way,” and “success rate” were translated literally. “watchful waiting” was translated into “watching and waiting,” and “permanently forgoing treatment” was translated literally.	“look for discussion of” was omitted, “the gaps” was translated into “lack,” “affects everyone in the same way” was translated into “applicable to all patients,” and “success rate” was translated into “100% effective.” “watchful waiting” was translated into “expecting treatment,” and “permanently forgoing treatment” was translated into “forgoing treatment” by omitting “permanently.”

^a^Italicization indicates problematic parts of the items in C-DISCERN.

^b^Ellipses stand for the omitted parts of the items that are not problematic.

#### Back Translation

In back translation, the qualified bilingual translator attached great importance to linguistic differences and cultural nuances between the Chinese and English languages. In this process, close attention was paid to linguistic and cultural appropriateness. For example, *a clear indication of* in back translated item 1, *a clear statement of* in back translated item 6, and *close attention* in back translated item 8 were used to cater to the linguistic tendency of using nominal expressions instead of verbal expressions. Similarly, *symptoms* and *recurrence* in item 10 and *side effects*, *complications*, and *adverse reactions* in item 11 were used to achieve cultural appropriateness in terms of core concepts, jargon, or technical terms. In addition, *date* rather than *time* was used in the back translation of item 5 to meet linguistic expectations in English. Syntactically, the active-voice clauses in Chinese items 4 and 5 were converted into passive-voice clauses in back translated items 4 and 5, respectively, to cater to habitual passivization in English. All these translation manipulations allowed the back translated version to be more idiomatic, as shown in Table 2.

**Table 2:** Exemplification of translation manipulations in back translation.

Original items	Back translated items
*1. Are the aims clear?*^a^*HINT:* Look for a clear indication at the beginning of the publication of: What it is about What it is meant to cover (and what topics are meant to be excluded) Who might find it useful	1. Is the purpose of this article clear? Look for content at the beginning of the article as *a clear indication of*^b^ the following questions: What is it about What may be covered or uncovered in it Who would find it useful
*4. Is it clear what sources of information were used to compile the publication (other than the author or producer)? HINT:* Check whether the main claims or statements made about treatment choices are accompanied by a reference to the sources used as evidence, eg, a research study or expert opinion. Look for a means of checking the sources used such as a bibliography or reference list or the addresses of the experts or organizations quoted, or external links to the online sources.	4. Does the article clearly state *what information was used*? Whether the sources of information referenced in the article regarding treatment options are explicitly mentioned, eg, the results of a study or the opinions of an expert Whether the sources of information are marked in the text, for example, references, addresses of cited experts or institutions, and links to online sources of information
*5. Is it clear when the information used or reported in the publication was produced? HINT:* Look for: Dates of the main sources of information used to compile the publication Date of any revisions of the publication (but not dates of reprinting in the case of print publications) Date of publication (copyright date)	5. Does the article clearly state *when the information used was published*? Please look for: Date of the main sources of information used to write this article Date of any revisions of the article (not dates of reprinting in the case of print publications) Date of publication (copyright dates)
*6. Is it balanced and unbiased? HINT:* Look for: A clear indication of whether the publication is written from a personal or objective point of view Evidence that a range of sources of information was used to compile the publication, eg, more than one research study or expert Evidence of an external assessment of the publication	6. Is the article objective, impartial, and unbiased? A clear statement of the writing angle: personal or objective A clear statement of various sources of information used in the text, such as several studies or opinions of several experts A clear statement of the external evaluation of the article
*8. Does it refer to areas of uncertainty? HINT:* Look for discussion of the gaps in knowledge or differences in expert opinion concerning treatment choices. Be wary if the publication implies that a treatment choice affects everyone in the same way, eg, 100% success rate with a particular treatment.	8. Does the article mention some kind of uncertainty? For example, there are controversies over knowledge and expert opinions on treatment options. Close attention: Does the article indicate that a treatment choice is suitable for everyone, eg, a particular treatment is 100% effective?
*10. Does it describe the benefits of each treatment? HINT:* Benefits can include controlling or getting rid of symptoms, preventing the recurrence of the condition, and eliminating the condition, both short-term and long-term.	10. Does the article describe the advantages of each treatment option? Including long-term and short-term control or elimination of *symptoms*, prevention of *recurrence*, or complete cure of the disease.
*11. Does it describe the risks of each treatment? HINT:* Risks can include side effects, complications, and adverse reactions to treatment, both short-term and long-term.	11. Does the article describe the risks of each treatment option? Including long-term and short-term *side effects*, *complications*, or *adverse reactions* to treatment.

^a^The original DISCERN instrument uses boldface to mark the questions for emphasis.

^b^Italicization was used in back translated items to mark translation manipulations.

#### Translation Equivalence Testing

By checking the back translated English version against the original English version, the native English speaker rated all back translated items with 1 or 2 in terms of SI but rated items 3, 4, 8, 9, and 15 with 3 or 4 in terms of CL, as presented in Table 3. When reviewing these seemingly problematic items rated negatively in CL, we found that they achieved a satisfactory rating of 2 in SI, although they were different from their counterparts in the original English version in syntactic or lexical choices. Such differences were acceptable when the linguistic appropriateness of English was warranted. Back translated items may, in theory, be different from the corresponding items in the original instrument in linguistic forms [34].

**Table 3:** Comparison of the back translated English version with the original English version in terms of similarity of interpretability (SI) and comparability of language (CL).

Original English version	Back translated English version	SI	CL
*1. Are the aims clear? HINT*^a^*:* Look for a clear indication at the beginning of the publication of: What it is about What it is meant to cover (and what topics are meant to be excluded) Who might find it useful	1. Is the purpose of this article clear? Look for content at the beginning of the article as a clear indication of the following questions: What is it about What may be covered or uncovered in it Who would find it useful	1	2
*2. Does it achieve its aims? HINT:* Consider whether the publication provides the information it aimed to as outlined in Question 1.	2. Did the article achieve its intended purpose? Consider whether the article provides the following information: What is it about What may be covered or uncovered in it Who would find it useful	1	2
*3. Is it relevant? HINT:* Consider whether: The publication addresses the questions that readers might ask Recommendations and suggestions concerning treatment choices are realistic or appropriate	3. Does the article address questions that readers may ask? Whether the treatment options available in the article are feasible and targeted	2	4
*4. Is it clear what sources of information were used to compile the publication (other than the author or producer)? HINT:* Check whether the main claims or statements made about treatment choices are accompanied by a reference to the sources used as evidence, eg, a research study or expert opinion. Look for a means of checking the sources used such as a bibliography or reference list or the addresses of the experts or organizations quoted, or external links to the online sources.	4. Does the article clearly state what information was used? Whether the sources of information referenced in the article regarding treatment options are explicitly mentioned, eg, the results of a study or the opinions of an expert Whether the sources of information are marked in the text, for example, references, addresses of cited experts or institutions, and links to online sources of information	2	3
*5. Is it clear when the information used or reported in the publication was produced?HINT:* Look for: Dates of the main sources of information used to compile the publication Date of any revisions of the publication (but not dates of reprinting in the case of print publications) Date of publication (copyright date)	5. Does the article clearly state when the information used was published? Please look for: Date of the main sources of information used to write this article Date of any revisions of the article (not dates of reprinting in the case of print publications) Date of publication (copyright dates)	1	2
*6. Is it balanced and unbiased? HINT:* Look for: • A clear indication of whether the publication is written from a personal or objective point of view • Evidence that a range of sources of information was used to compile the publication, eg, more than one research study or expert • Evidence of an external assessment of the publication	6. Is the article objective, impartial, and unbiased? • A clear statement of the writing angle: personal or objective • A clear statement of various sources of information used in the text, such as several studies or opinions of several experts • A clear statement of the external evaluation of the article	2	2
*7. Does it provide details of additional sources of support and information? HINT:* Look for suggestions for further reading or for details of other organizations providing advice and information about the condition and treatment choices.	7. Does the article detail other sources of support and information? Look in the text for suggestions for further reading or other organizations that provide advice and information on conditions and treatment options.	1	1
*8. Does it refer to areas of uncertainty? HINT:* Look for discussion of the gaps in knowledge or differences in expert opinion concerning treatment choices. Be wary if the publication implies that a treatment choice affects everyone in the same way, eg, 100% success rate with a particular treatment.	8. Does the article mention some kind of uncertainty? For example, there are controversies over knowledge and expert opinions on treatment options. Close attention: Does the article indicate that a treatment choice is suitable for everyone, eg, a particular treatment is 100% effective?	2	3
*9. Does it describe how each treatment works?HINT:* Look for a description of how a treatment acts on the body to achieve its effect.	9. Does the article describe the specific operations of each treatment? Please look for a description in the text that a certain treatment works and produces curative effects in the human body.	2	3
*10. Does it describe the benefits of each treatment? HINT:* Benefits can include controlling or getting rid of symptoms, preventing the recurrence of the condition, and eliminating the condition, both short-term and long-term.	10. Does the article describe the advantages of each treatment option? Including long-term and short-term control or elimination of symptoms, prevention of recurrence, or complete cure of the disease.	1	2
*11. Does it describe the risks of each treatment? HINT:* Risks can include side effects, complications, and adverse reactions to treatment, both short-term and long-term.	11. Does the article describe the risks of each treatment option? Including long-term and short-term side effects, complications, or adverse reactions to treatment.	1	1
*12. Does it describe what would happen if no treatment is used? HINT:* Look for a description of the risks and benefits of postponing treatment, of watchful waiting (ie, monitoring how the condition progresses without treatment), or of permanently forgoing treatment.	12. Does the article describe the consequences of not taking any treatment? Look in the text for descriptions of the risks and benefits of delaying treatment, expecting treatment (to watch the disease progress), or forgoing treatment forever.	2	2
*13. Does it describe how the treatment choices affect the overall quality of life? HINT:* Look for: Description of the effects of the treatment choices on day-to-day activity Description of the effects of the treatment choices on relationships with family, friends, and carers	13. Does the article describe the impact of treatment options on quality of life? The article describes the impact of treatment options on daily life, as well as on the patient’s relationship with family, friends, and caregivers.	1	1
*14. Is it clear that there may be more than one possible treatment choice? HINT:* Look for: A description of who is most likely to benefit from each treatment choice mentioned and under what circumstances Suggestions of alternatives to consider or investigate further (including choices not fully described in the publication) before deciding whether to select or reject a particular treatment choice	14. Does the article provide more than one treatment option? Whether the article mentions the patient population for which each treatment option is appropriate and in what circumstances it would be helpful to the patient Whether the article proposes other alternative treatment options, including those not adequately described in the article, before the patient decides to choose or forgo a treatment option	1	2
*15. Does it provide support for shared decision-making? HINT:* Look for suggestions of things to discuss with family, friends, doctors, or other health professionals concerning treatment choices.	15. Did the article help the patient’s family, friends, doctor, or other medical staff work together to choose the best treatment option for the patient?	2	4
*16. Based on the answers to all of the above questions, rate the overall quality of the publication as a source of information about treatment choices.*	16. Please rate the overall quality of the article as a source of information on treatment options based on your answers to all of the above questions.	1	2

^a^The original DISCERN instrument uses italicization to mark the questions for emphasis.

Another apparent difference between these 2 English versions was whether boldface was used. As boldface is seldom used for emphasis in Chinese, the Chinese version did not use this technique, resulting in a lack of boldface in the back translated English version. However, this disparity did not cause any difference in SI and CL, especially considering the separation of the question items from the hints following them in both English versions. On the basis of the results of the review, we concluded that items 3, 4, 8, 9, and 15 achieved semantic equivalence and communicated the original meaning and intent of the corresponding original items regardless of their low level of CL. Therefore, we did not further improve the SI and CL of C-DISCERN.

### Psychometric Property Testing

#### Content Validation

According to the 3 health educators’ ratings of the content of C-DISCERN, it had a content validity index of 0.98 (47/48, 98% of the items) for clarity and of 0.94 (45/48, 94% of the items) for relevance. One health educator rated all 16 items with 3 or 4 for both clarity and relevance. Item 3 was rated with 2 for both clarity and relevance by 1 rater. Item 4 was rated with 2 for relevance by 1 rater. Item 15 was rated with 1 for relevance by 1 rater. The remaining items were rated with 3 or 4 by all 3 raters. These seemingly problematic items were subjected to the final judgment of a highly qualified health educator, who found that these items were clear and relevant. As a result, they were not revised.

#### Reliability Testing

##### Interrater Reliability

Through statistics of the 2 health educators’ ratings of the selected materials, the Cohen κ coefficient was calculated at 0.53 (95% CI 0.417-0.698), indicating a moderate interrater agreement according to Fleiss [55]. This meant that C-DISCERN was reliable in terms of the scoring agreement between independent raters.

##### Internal Consistency

We obtained a Cronbach α value of .93 (95% CI 0.699-1.428) for the internal consistency of C-DISCERN. This result showed that the construct of C-DISCERN had good internal consistency [56,57].

## Discussion

### Principal Findings

Although it is difficult to make a tool culturally relevant, comprehensible, and faithful to the original meaning through linguistic and cultural adaptation [50], we successfully translated and adapted the DISCERN instrument into C-DISCERN and verified its validity and reliability for assessing the quality of health information on treatment choices. A total of 4 strategies adopted in the entire translation and adaptation process ensured the semantic equivalence and cultural appropriateness of C-DISCERN, including forward translation, semantics evaluation and consolidation of the translated version, back translation, translation equivalence testing, and further adaptation. Hopefully, this newly developed instrument can be effectively applied to save health information providers’ time in screening, introduce more reliability to the quality of consumer health information produced and distributed, improve the communication and shared decision-making skills of health professionals, and help patients make decisions on treatment.

The choice of qualified bilingual translators was the prerequisite for quality translation. We fully considered 1 fact before selecting translators for this study: qualified translators are not always adequately knowledgeable in specialized subject areas related to some scales and are frequently incapable of translating the content area of medical materials [34]. The translator we used was a qualified bilingual translator who had relatively rich experience in engaging in the translation and translation studies of health and medical materials, warranting the quality of both forward and back translation.

In back translation, we were allowed to modify words or concepts that had no equivalent in the other language [33]. We tried to retain the same meaning of the 16 items of DISCERN after they were translated into simplified Chinese, which was the key to ensuring semantic equivalence [59]. Back translation also facilitated the achievement of conceptual equivalence, strengthening the reliability of the newly developed instrument and enhancing the validity of this study and the credibility of the findings [33].

Although back translation was used to verify the translation of DISCERN, the target-language version may be inappropriate for use in the target population [33]. Considering this possible inappropriateness, we performed a systematic comparison both between the source-language version and the target-language version and between the original English version and the back translated English version, informed by Tang and Dixon [60]. Systematic comparison and corresponding revisions effectively improved the cultural appropriateness of C-DISCERN.

In addition to the aforementioned effective methods of translation, we adopted some methods of testing C-DISCERN among monolingual Chinese-speaking participants, which is imperative to validate the clarity and appropriateness (relevance) of the target-language version [33]. To this end, we tested the psychometric properties of C-DISCERN, including its content validity, interrater agreement (reliability), and internal consistency. It is a common practice to comprehensively evaluate the psychometric properties of any newly developed tool [33].

### Comparison With Previous Studies

Translation is a challenging task, calling for skill, knowledge, and experience [34]. Brislin et al [46] argued that even professional translators cannot eliminate critical translation problems that affect many studies negatively. This is owing to the difficulty in finding qualified bilingual translators knowledgeable in the content and subject areas of the instruments that need to be translated [22,48]. In addition, rigorous translation procedures, cultural nuances, jargon, idiomatic phrases, and emotionally evocative words [34] all make the already challenging translation task even more complicated. To address these difficulties, we not only chose translators carefully but also implemented translation and adaptation strategies rigorously. The forward translator conveyed the original meanings and intents successfully by choosing culturally equivalent linguistic expressions [34]. Back translation helped us detect problem items [48,61].

Although time-consuming, back translation is highly recommended as an effective strategy for the translation of study instruments, as claimed by Sperber [34]. However, this technique may involve some traps. Capable translators can produce a back translated text similar to the source text even when the forward translated text is not satisfactory [62]. When compared with the source text, such a back translation would exhibit a high level of SI and CL, most likely to cover the discrepancies between the source text and the target text. In this case, a systematic comparison proposed by Tang and Dixon [60] should be conducted not only to compare the target-language text with the source-language text but also to compare the back translated text with the source text. This type of systematic comparison was used in this study to perform translation equivalence testing and further adaptation, making the translated scale more culturally and linguistically appropriate.

SI and CL proposed by Sperber et al [50] were helpful indicators that we used to check the back translated English version against the original English version of DISCERN. All the translated items achieved a high level of SI. Items rated positively for SI but negatively for CL were not revised as we pursued semantic equivalence rather than formal equivalence. Although taking different linguistic forms, back translated items rated poorly for CL were still loyal to the original items. This priority given to SI confirmed the finding in the literature that “similarity of meaning, even at the expense of similarity of form, is much more desirable than the opposite” [34]. The form can be purposefully changed to ensure equivalence of meaning [34]. To guarantee semantic equivalence, we adopted some translation techniques such as addition, deletion, substitution, and omission, changing the form of the original text. We found these strategies effective in this study, contrary to the finding of Sperber [34], who described these methods as common translation errors.

The content validity index of C-DISCERN was determined at 0.98 (47/48, 98% of the items) for clarity and 0.94 (45/48, 94% of the items) for relevance. We could not compare these indexes with those reported in previous studies to identify factors possibly affecting content validity as this study is the only one describing the content validity of the translated DISCERN. The high level of content validity in this study can be attributed to two factors: (1) the health educators’ knowledge of and expertise in health materials, which facilitated their understanding of C-DISCERN, and (2) the conceptual equivalence, reliability, and validity of C-DISCERN resulting from rigorous translation and adaptation procedures. Future studies need to pay attention to the content validity of newly developed instruments through translation and adaptation. Low levels of content validity indexes would affect the reliability of new scales.

C-DISCERN demonstrated a moderate interrater agreement (Cohen κ coefficient=0.53, 95% CI 0.417-0.698). This finding is consistent with those of previous studies. Batchelor and Ohya [63] rated brochures and websites on eczema and asthma using the Japanese DISCERN, finding an average Cohen κ value of 0.53 (95% CI 0.13 to 0.18) among experts. McCool et al [3] investigated the reliability of DISCERN for assessing the quality of information available to German patients with eczema, obtaining a Cohen κ value of 0.59 (95% CI 0.477-0.709). Logullo et al [28] translated DISCERN into Brazilian Portuguese and tested its psychometric properties, obtaining a Cohen κ value of 0.85 (95% CI 0.717-0.912) for interrater agreement. Montoya et al [29] merely translated DISCERN into Spanish and rated the comparability, interpretability, and understandability of the translated tool without testing its psychometric properties. Dierks et al [30] only translated DISCERN into German without describing its content validity and psychometric properties. From the aforementioned studies reporting interrater agreement, DISCERN translated and adapted into other languages exhibited fair to nearly complete interrater agreement. The discrepancies in Cohen κ values revealed that raters’ health and medical qualifications seemed to be adversely proportional to the interrater agreement: McCool et al [3], Batchelor and Ohya [58], and this study all invited health educators or experts as raters, each resulting in a Cohen κ value for moderate interrater agreement, whereas Logullo et al [28] used journalism students as raters, obtaining a Cohen κ value for nearly complete interrater agreement. However, we can only tentatively draw this conclusion, which needs to be further verified in future research. This is because this conclusion is inconsistent with the finding of Charnock et al [9] that the Cohen κ values for experts, information providers, and patients were 0.53, 0.40, and 0.23, respectively. The finding of Charnock et al [9] seemed to hint at a positively proportional relationship between medical and health knowledge and expertise and interrater agreement. This positive proportional relationship was supported by previous studies that clearly stated that the Cohen κ value disparities resulted mainly from the varying experiences of raters in assessing health education materials [22,64,65]. Moreover, the disparities in interrater agreement stemmed also from different degrees of rater subjectivity, as found by Weintraub et al [66]. Another possible factor could be the degree of subjectivity of the rating criteria of instruments: more subjectivity of rating criteria can cause lower interrater agreement [22,64]. We assumed that these last 2 factors may also have affected the internal consistency of the translated DISCERN instrument.

In this study, C-DISCERN displayed a high level of internal consistency (Cronbach α=.93, 95% CI 0.699-1.428). Similarly, the Portuguese DISCERN showed a high degree of internal consistency (Cronbach α=.87, 95% CI 0.826-0.898) in Logullo et al [28], and the German DISCERN had a high level of internal consistency (Cronbach α=.81, 95% CI 0.753–0.964) in McCool et al [3]. Other studies that were related to the translation and validation of DISCERN failed to investigate the internal consistency of the translated DISCERN [29,30,63]. Various previous studies [16-21,23,24] used DISCERN to directly assess the quality of health and medical materials, but they did not investigate the internal consistency given that DISCERN has already been validated. A positive proportional relationship between medical and health knowledge and expertise and interrater agreement does not seem applicable to the internal consistency of the translated DISCERN tool, as indicated by the aforementioned values of the Cronbach α. Thus, the influence of the subjectivity of the raters may be a contributing factor. There was “latitude allowed in the interpretation of the criteria” that possibly resulted in subjectivity in rating health education materials [66].

The features of the DISCERN versions used in previous translation and validation studies are presented in Table 4. Although these studies did not report the limitations induced by the subjective evaluation of the raters of consumer health education information, we concluded that the raters’ different degrees of subjectivity could cause differences in interrater agreement, as reported by Weintraub et al [66] that there was “latitude allowed in the interpretation of the criteria” that possibly led to subjectivity in rating health education materials. As a result, we propose that training programs be conducted to enrich raters’ experience in assessing health education materials to improve interrater reliability [28].

**Table 4:** Features of DISCERN versions translated and validated in different languages.

DISCERN version	Population in which the DISCERN versions were validated	Materials used	Raters	Limitations induced by subjective evaluation
German; McCool et al [3]	2 independent raters with backgrounds in public health and medicine	Information available to German patients with eczema	2 independent raters with backgrounds in public health and medicine	Unspecified
Portuguese; Logullo et al [24]	126 journalism students	A text about smoking cessation treatments	126 journalism students	Unspecified
Spanish; Montoya et al [25]	20 raters fluent in the source language (English)	No	20 raters fluent in the source language (English)	Unspecified
German; Dierks et al [26]	15 physicians	No	15 physicians	Unspecified
Japanese; Batchelor and Ohya [59]	15 members of the medical staff and 9 carers of 9 children attending the Department of Allergy outpatient clinic at the National Center for Child Health and Development, Japan	Information contained in pamphlets on treatments for asthma and atopic dermatitis	15 members of the medical staff and 9 carers of 9 children	Unspecified

### Limitations

This study has several limitations. First, we could not eliminate all potential linguistic and cultural nuances in the cross-cultural translation of DISCERN, especially with respect to the communication of some peculiar living experiences specific to the source language and culture. However, we conducted rigorous translation, adaptation, and validation, ensuring maximal semantic equivalence, cultural appropriateness, and validity and reliability of C-DISCERN. Second, we only ascertained the validity and reliability of C-DISCERN for assessing the quality of a limited number of lung cancer–related health materials on treatment choices in this study. Its applicability to a large number of patient-targeted health materials on the choices of treatment for other diseases still needs to be verified in future research. Third, this newly developed tool has been proven valid and reliable only from the perspective of health educators. Its validity and reliability need to be attested by patients in future research. Future studies need to be conducted to attest to the validity of this newly developed instrument for assessing other health education materials from the perspective of patients and the public. Fourth, we compared C-DISCERN only with a limited number of scales in languages other than Chinese that have been translated and adapted from the original DISCERN instrument in terms of content validity, interrater agreement, and internal consistency. This restricted comparison prevented us from identifying other possible factors that may influence the validity and reliability of newly developed assessment tools through translation and adaptation. Finally, future studies should involve informants with diverse education levels, age ranges, health literacy levels, socioeconomic backgrounds, and psychological health states to reveal the influence of these factors on the study results.

### Conclusions

C-DISCERN is the first simplified Chinese version of the DISCERN instrument. It has been verified as a valid and reliable scale for evaluating the quality of lung cancer–related patient information. This newly developed instrument is intended to assess the quality of patient-targeted information on treatment choices. It has a high potential to be used as an effective assessment tool for health information in print and digital texts, especially given the fact that there are few health information assessment measures currently available in mainland China. It can allow health educators and health care providers to select quality health education materials on treatment choices for health education and interventions. It can also inform developers of health education materials on treatment choices to effectively promote the understandability and actionability of such information. These efforts will be hopefully conducive to patients’ immediate behavior changes, favorable medical actions, and desired health outcomes.

## Abbreviations

C-DISCERN: simplified Chinese version of the DISCERN instrument
CL: comparability of language
PEMAT: Patient Education Materials Assessment Tool
SAM: Suitability Assessment of Materials
SI: similarity of interpretability
